# Fluid Resuscitation and Initial Management in Patients Presenting with Sepsis in the General Ward

**DOI:** 10.3390/life15010124

**Published:** 2025-01-18

**Authors:** Sung Won Chang, Juwhan Choi, Jee Youn Oh, Young Seok Lee, Kyung Hoon Min, Gyu Young Hur, Sung Yong Lee, Jae Jeong Shim, Jae Kyeom Sim

**Affiliations:** Division of Pulmonary, Allergy, and Critical Care Medicine, Department of Internal Medicine, Korea University Guro Hospital, Korea University College of Medicine, Seoul 08308, Republic of Korea; lego42st@gmail.com (S.W.C.); syl0801@korea.ac.kr (S.Y.L.);

**Keywords:** sepsis, general ward, fluid, resuscitation

## Abstract

The optimal management of hospital-presenting sepsis remains poorly understood. We investigated the initial management in patients presenting with sepsis in the general ward, the association between fluid resuscitation and clinical outcomes, and the factors affecting fluid resuscitation. A retrospective study was conducted on patients who presented with sepsis-induced hypotension in the general ward. Patients were divided into Less 30 (fluid resuscitation less than 30 mL/kg) and More 30 (fluid resuscitation 30 mL/kg or more) groups. Multivariable logistic regression analysis was performed. The median resuscitation fluid volume was 500 mL (9.2 mL/kg) and 2000 mL (35.9 mL/kg) in the Less 30 (*n* = 79) and More 30 (*n* = 11) groups, respectively. The intensive care unit (ICU) mortality was similar between the two groups (43.0% vs. 45.5%). Twenty-two patients received continuous renal replacement therapy (CRRT) in the Less 30 group, whereas none received it in the More 30 group (27.8% vs. 0%). Fluid resuscitation ≥30 mL/kg was not associated with ICU mortality. Low body weight and systolic blood pressure were associated with fluid resuscitation ≥30 mL/kg. Most hospital-presenting sepsis patients received less than 30 mL/kg of fluid, and fluid resuscitation was not associated with ICU mortality.

## 1. Introduction

Sepsis and septic shock are medical emergencies where early recognition and treatment have a significant effect on prognosis. Fluid resuscitation is an essential component of early management, and the 2016 Surviving Sepsis Campaign guidelines recommend that at least 30 mL/kg of intravenous crystalloid be given within the first 3 h. The importance of fluid resuscitation was also noted in the 2021 guidelines [1,2]. While the significance of the early management of sepsis is well established in emergency departments and intensive care units (ICUs), it is less recognized in the general ward [3].

Approximately 10 to 20% of sepsis and septic shock occur in hospitalized patients (hospital-presenting sepsis), and their mortality is much higher than that of patients presenting with sepsis at the time of admission [4,5]. Patients with hospital-presenting sepsis have more comorbidities than those with community-onset sepsis [5]. Adherence to the sepsis bundle, such as early blood cultures and antibiotic administration, is lower in these patients [6]. Suboptimal fluid resuscitation may also contribute to poor patient outcomes. Observational studies comparing fluid resuscitation in patients with hospital-presenting sepsis and patients with emergency department-presenting sepsis showed that fluid administration was initiated in a timely manner in 40% of hospitalized patients, in contrast to 78% of patients in the emergency department [7]; the administered volume was lower by 10 mL/kg in hospitalized patients than those in the emergency department [8].

Notably, renowned clinical trials forming the basis of resuscitation strategies have been conducted in the emergency department [9,10,11,12]. Hospitalized patients were not included in any previous study on fluid resuscitation during septic shock [13]. Therefore, the effect of fluid resuscitation in hospitalized patients remains unclear. These patients may be vulnerable to fluid overload because of comorbidities, such as congestive heart failure, renal failure, or advanced liver disease [5,8,14]. Because a significant proportion of hospitalized patients already receive maintenance fluid therapy [15,16], they may be less hypovolemic or less responsive to fluid resuscitation at the time of septic shock. Conversely, clinicians may not provide adequate fluid resuscitation despite recognizing sepsis or septic shock given the clinical context, including the concerns mentioned above [6,17,18].

We hypothesized that fluid resuscitation is not sufficiently provided to hospitalized patients and that less fluid resuscitation leads to poor clinical outcomes. Therefore, we investigated the initial management of patients presenting with sepsis in the general ward, including fluid resuscitation, and the association between the resuscitation fluid volume and patient outcomes. Additionally, we investigated the factors affecting resuscitation fluid volume.

This article is a revised and extended version of a paper entitled ‘Fluid resuscitation and initial management in patients presenting sepsis on general ward’, which was presented at 27th Congress of the Asian Pacific Society of Respirology in Singapore on 17 November 2023 [19].

## 2. Materials and Methods

### 2.1. Study Design and Population

This is a retrospective, observational study conducted in a university-affiliated hospital in South Korea. We collected data of patients who were transferred from the general ward to the medical ICU from August 2016 to July 2021. We included patients who were 19 years or older, presented with sepsis in the general ward, and were transferred to the medical ICU. In our hospital, intensivists manage the admission and discharge of patients in ICU. Attending physicians in general wards briefly document reasons for ICU admission and make referrals. We used these admission referral records to identify patients whom attending physicians clinically judged to have sepsis. Patients were excluded based on the following criteria: (1) admission to the ICU from the emergency department, (2) admission to the surgical ICU, and (3) admission to the ICU for reasons other than sepsis. This study was approved by the Institutional Review Board of Korea University Guro Hospital (2022GR0521). This study was conducted in accordance with the Declaration of Helsinki. The requirement for informed consent was waived as it was an observational study. We ensured patient privacy and anonymity during the study.

### 2.2. Study Definition

Patients were considered to have sepsis if they met all three of the following criteria: (1) having a proven or suspected infection, (2) being hypotensive, and (3) receiving vasopressor. Hypotension was defined as systolic blood pressure < 90 mmHg or mean blood pressure < 65 mmHg. These patients were compatible with sepsis as defined by Sepsis-3. As lactate levels were often not readily available for patients on general wards, we did not further differentiate septic shock [20]. Time zero was defined as the first time that the hypotension criteria were met in patients with sepsis, as previously described.

### 2.3. Data Collection

Data regarding baseline characteristics, initial sepsis management, and clinical outcomes were collected from medical records. Initial sepsis management included fluid resuscitation, vasopressor administration, albumin administration, blood cultures, and newly administered antibiotics. Based on time zero, the time point at which each management was performed was calculated. The total resuscitation fluid volume was the amount of rapidly infused crystalloid (fluid bolus) for the correction of hypotension within the first 3 h from time zero. The amount of other fluids, such as of maintenance fluid, replacement fluid, nutritional fluid, or fluid creeps was not considered [21]. Because it was difficult to obtain the exact completion time, and only the fluid bolus was measured, the fluid resuscitation start time was used for the calculation. Newly administered antibiotics were defined as the first antibiotic administered to antibiotic-naïve patients or antibiotics added or changed to patients already on antibiotic treatment on the day of sepsis onset. The time at which antibiotics and blood cultures were ordered was used for the time point calculation because the exact time of execution was difficult to measure. The primary outcome of interest was ICU mortality. We also examined in-hospital mortality, ICU and hospital length of stay, and the use of mechanical ventilation and CRRT. Only the first admission was included in the analysis of patients with multiple ICU admissions during the study period.

### 2.4. Statistical Analysis

Patients were divided into the Less 30 (<30 mL/kg) and More 30 (≥30 mL/kg) groups according to the total resuscitation fluid volume per body weight within the first 3 h. Categorical variables were reported as numbers and percentages and compared using Fisher’s exact or Chi-square tests. Continuous variables were reported as medians with interquartile ranges and compared using the Mann–Whitney *U*-test. Multivariable logistic regression analysis was performed to examine the association between fluid resuscitation and ICU mortality. We also investigated the factors associated with fluid resuscitation ≥30 mL/kg. Baseline characteristics and initial sepsis management were utilized as variables in the logistic regression analysis. Variables with a *p* value < 0.1 in univariable analysis were included in the multivariable analysis using the backward elimination method. The results were reported with the odds ratio (OR) of each variable and a 95% confidence interval (CI). All tests were two-sided, and a *p* value < 0.05 was considered statistically significant. All statistical analyses were performed using SPSS (version 20.0; IBM Corp., Armonk, NY, USA).

## 3. Results

**Figure 1:**
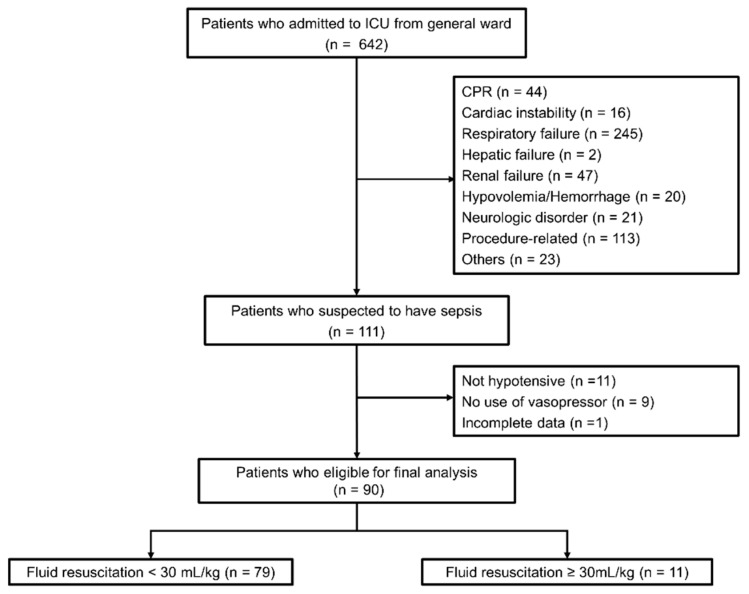
Flowchart of patient identification. ICU, intensive care unit; CPR, cardiopulmonary resuscitation.

During the study period, 642 patients were transferred from the general ward to the medical ICU. Among them, 111 were suspected of having sepsis by the attending physicians. Based on the study definitions, 90 patients were included in the final analysis. 79 patients received < 30 mL/kg of fluid resuscitation within first the first 3 h (Less 30 group), and 11 patients received at least 30 mL/kg (More 30 group) (Figure 1).

### 3.1. Baseline Characteristics

Overall, the baseline characteristics were similar in both groups (Table 1). The median age was 66.5 years. The most common underlying disease was malignancy; almost half of patients had solid cancers and more than one-quarter had hematological malignancies. The distribution of infection foci was similar in both groups, with pneumonia and intra-abdominal infection being the most common. The median hospital length of stay before the onset of sepsis was 13 days. A total of 60% of patients were already receiving antibiotics before sepsis onset. Body weight and systolic blood pressure at time zero were lower in the More 30 group (57.4 kg vs. 47.2 kg, *p* = 0.005 and 80 mmHg vs. 71 mmHg, *p* = 0.015, respectively) than in the Less 30 group. We considered mean blood pressure < 65 mmHg as the criterion for hypotension; however, mean blood pressure was only ascertainable in four participants of the study. Hence, it was not analyzed. The More 30 group tended to receive less oxygen therapy; however, there was no statistical significance (55.7% vs. 27.3%, *p* = 0.077).

### 3.2. Initial Sepsis Management Within 3 Hours from Time Zero

Fluid resuscitation and other management profiles are presented in Table 2 and Table S1. The total resuscitation fluid volume within 3 h from time zero was 500 mL and 2000 mL in each group. The fluid volume per body weight was 9.2 mL/kg and 35.9 mL/kg, respectively. The median time to fluid resuscitation was similar in both groups (2 min vs. 0 min, *p* = 0.068). Albumin was administered to 22.2% of all patients. All received 100 mL of 20% albumin, except one who received 200 mL of 20% albumin. The proportion of patients who received vasopressor within 3 h was similar (68.4% vs. 72.7%, *p* > 0.999). The median time taken to initiate vasopressor administration was 123 min and 90 min in the Less 30 and More 30 groups, respectively, without statistical significance (*p* = 0.160). Blood cultures were obtained from 15.6% of all patients within 3 h from time zero. Approximately 31% of all patients received new antibiotics within 3 h, and the administration of new antibiotics within 3 h was twice as high in the More 30 group as in the Less 30 group, but it was not statistically significant (27.8% vs. 54.5%, *p* = 0.090). The median time to administer new antibiotics and to obtain blood cultures from time zero was shorter in the More 30 group.

**Figure 2:**
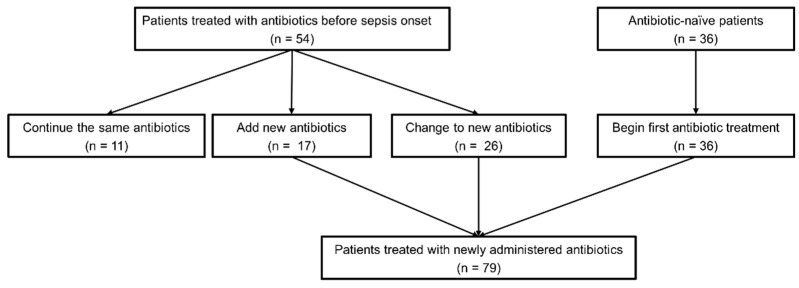
Prescription patterns of antibiotics.

In a significant number of patients, blood cultures and new antibiotic administrations were performed before time zero. Blood cultures were performed 1 or 2 days before the day of sepsis onset in 20 patients. Blood cultures were obtained on the day of sepsis onset from 63 patients, more than half of which were obtained before time zero (Table S2). Among the 54 patients who received antibiotics before the day of sepsis onset, 26 changed antibiotics on the day of sepsis onset. New antibiotics were added to 17 of the remaining 28 patients. Thirty-six patients received the first antibiotic treatment on the day of sepsis onset. Consequently, new antibiotics were administered to 79 patients on the day of sepsis, 31 before time zero, and 48 after time zero (Figure 2 and Table S3).

### 3.3. Clinical Outcomes

**Figure 3:**
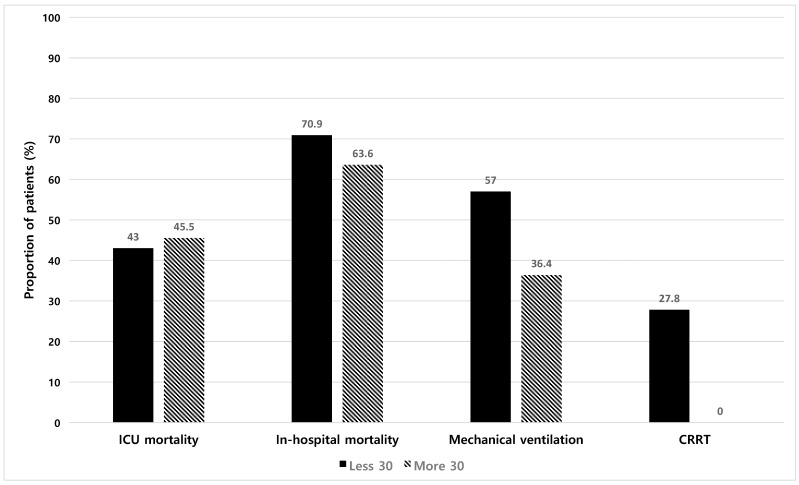
Comparison of clinical outcomes. ICU, intensive care unit; CRRT, continuous renal replacement therapy.

The clinical outcomes were comparable between both groups (Table 3) (Figure 3). The ICU mortality was 43% in all patients and did not differ between the groups (43.0% vs. 45.5%, *p* > 0.999). The overall in-hospital mortality was high at 70%. Twenty-two patients in the Less 30 group received continuous renal replacement therapy (CRRT), whereas none received it in the More 30 group (27.8% vs. 0%, *p* = 0.059). Of note, among those who received CRRT, no one had underlying chronic kidney disease or end-stage renal disease.

### 3.4. Factors Associated with ICU Mortality

**Figure 4:**
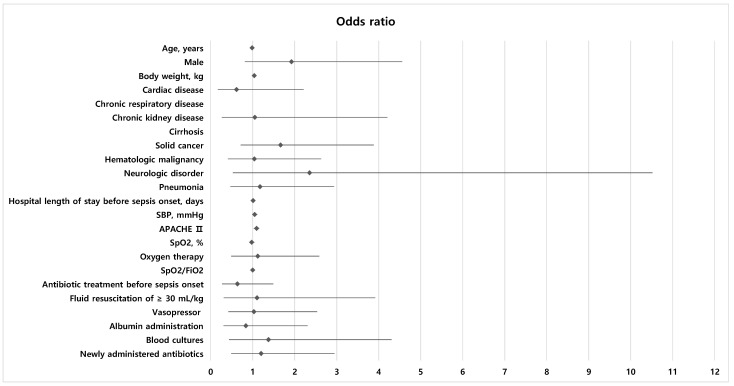
Forest plot displaying the odds ratios and 95% confidence interval of each factor for ICU mortality. For body weight and APACHE II, values obtained through multivariable analysis are displayed in the graph. SBP, systolic blood pressure; APACHE II, Acute Physiology and Chronic Health Evaluation II; SpO_2_, oxygen saturation; FiO_2_, fraction of inspired oxygen.

The results of the logistic regression analysis for ICU mortality are shown in Table 4 and Figure 4. Body weight and the Acute Physiology and Chronic Health Evaluation II score were included in the multivariable analysis. Because the effect of fluid resuscitation was the primary variable of interest in this study, fluid resuscitation ≥30 mL/kg was also included in the multivariable analysis, despite its *p* value being >0.1. After all, only the Acute Physiology and Chronic Health Evaluation II score was associated with ICU mortality (OR = 1.090, 95% CI = 1.028–1.156, *p* = 0.004).

### 3.5. Factors Associated with Fluid Resuscitation ≥30 mL/kg

**Figure 5:**
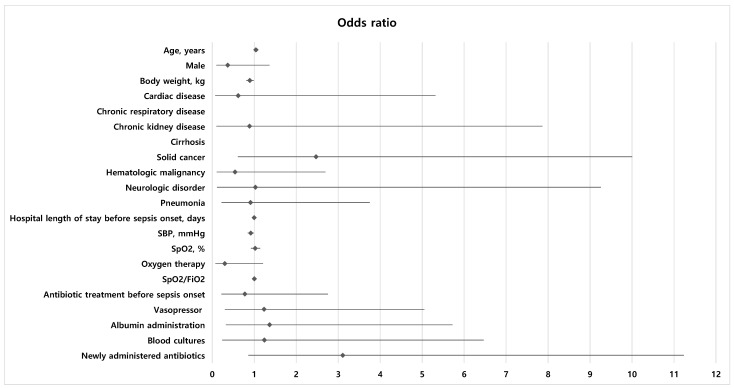
Forest plot displaying the odds ratios and 95% confidence interval of each factor for fluid resuscitation ≥30 mL/kg. For body weight and SBP, values obtained through multivariable analysis are displayed in the graph. SBP, systolic blood pressure; SpO_2_, oxygen saturation; FiO_2_, fraction of inspired oxygen.

Among baseline characteristics, systolic blood pressure and body weight were associated with fluid resuscitation ≥30 mL/kg (OR = 0.896, 95% CI = 0.811–0.989, *p* = 0.030 and OR = 0.916, 95% CI = 0.846–0.992, *p* = 0.031, respectively). Oxygen therapy and newly administered antibiotics were included in the multivariable analysis, but they were found to be non-significant factors (Table 5) (Figure 5).

## 4. Discussion

In this study, the median resuscitation fluid volume was 10.5 mL/kg and only 12% of the patients received fluid ≥ 30 mL/kg, which is consistent with our first hypothesis. However, contrary to the second hypothesis, there was no difference in clinical outcomes including ICU mortality between the Less 30 and More 30 groups. Fluid resuscitation ≥30 mL/kg was not associated with ICU mortality in the multivariable analysis. However, CRRT use tended to be lower in the More 30 group.

Providing an ample amount of fluid has long been regarded as the core element of sepsis management. Although hospital-presenting sepsis has received relatively little attention, several meaningful studies on the subject have recently been conducted. One study showed that an increased initial fluid volume was associated with decreased mortality in hospital-presenting sepsis [8]. Therefore, it is worthwhile to reconsider the findings of our study, in which fluid resuscitation was not related to ICU mortality. In another study, mortality was lowest in patients who received 20–35 mL/kg of fluid within the first 6 h and was higher in those who received a smaller volume of fluid. Mortality was also higher in patients who received > 35 mL/kg of fluid, although the difference was not statistically significant. Time taken to initiate fluid resuscitation was another factor affecting mortality, with lower mortality in patients with earlier initiation [7]. In our study, the median resuscitation fluid volume was 35.9 mL/kg in the More 30 group and 9.2 mL/kg in the Less 30 group during the first 3 h. If the timeframe was extended to the first 6 h, the administered fluid volume would increase. Then, in the More 30 group, it would exceed 35 mL/kg even more, while in the Less 30 group, it would become closer to 25–30 mL/kg. Additionally, the time taken to initiate fluid resuscitation was short in our study. The effect of the difference in crystalloid volume might be diluted because 22% of the study population received albumin. On the other hand, fluid resuscitation may not have a greater impact on clinical outcomes than expected. Intravenous fluid treatment was not associated with mortality among patients with hospital-presenting sepsis, as they may have been more volume replete [22,23]. Even in patients with community-onset sepsis, the administration of ≥30 mL/kg of fluid within the first 6 h was not associated with mortality [24,25]. Notably, the latest 2021 Surviving Sepsis Campaign guidelines have downgraded the recommendation strength for fluid resuscitation [2].

We identified low blood pressure and body weight as factors related to fluid resuscitation of ≥30 mL/kg. Low blood pressure has long been the most common indication for fluid bolus [26]. Low body weight is a well-known predictor of fluid resuscitation. Previous studies have shown that obese patients have lower odds of receiving ≥ 30 mL/kg of fluid within 3 h, and a higher body mass index is associated with lower fluid volume per body weight [27,28]. Presumably, most clinicians administer fluid in fixed volume (i.e., 500 mL) boluses.

Interestingly, only patients in the Less 30 group were supported by CRRT. However, this finding should be interpreted with caution. It may demonstrate the positive effect of ≥30 mL/kg of fluid resuscitation; on the other hand, it may be that less fluid was given to patients at risk of volume overload or oliguria due to renal impairment [27].

Another notable finding of this study was that antibiotic treatment and blood cultures were performed in many patients before the day of sepsis onset or time zero. Although it is recommended that sepsis bundles be executed at the time of sepsis recognition, it is challenging to recognize sepsis in general wards [18]. Moreover, clinicians may order antibiotics and blood cultures in response to signs of infection, such as fever, rather than recognize sepsis and then take appropriate action. It may be reasonable to assess and treat infection before the obvious manifestation of sepsis. It is worthwhile to consider the correct approach when hypotension or other organ dysfunction occurs in patients with signs of infection, as in our study population. In previous studies, patients previously treated with antibiotics were considered to have completed sepsis bundles or were excluded from the study. However, the selection of a study population in that way may not fully reflect the complexity of clinical practice [22,29]. It is unclear whether antibiotics should be changed or added to broaden antibacterial coverage, whether blood cultures should be repeated, or whether only hemodynamic support should be provided.

This study has strengths in that it examined not only fluid resuscitation but also the administered dose and analyzed the pattern of antibiotic treatment and blood culture in detail. However, this study had several limitations. First, the sample size was small. We explain the causes of the lack of association between fluid resuscitation and mortality; however, low statistical power may be the reason. Second, this study has inherent limitations as a retrospective investigation. Patient selection for sepsis was based on medical records, requests for admission to the ICU, and pre-defined criteria; however, there may be potential selection bias. Although multivariable analysis was employed to assess the association between fluid resuscitation and mortality, it is not possible to exclude unmeasured or unadjusted confounding factors. In particular, as patients in general wards were treated at the discretion of attending physicians, rather than following a defined protocol, caution is needed when interpreting the effect of a single intervention (fluid resuscitation). Third, this study was conducted at a single center and predominantly included patients with malignancy, which limits the generalizability of our findings. We excluded reasons that could cause shock or require admission to the ICU as far as possible. As a result, the number of patients with other chronic diseases seemed to have decreased. Fourth, we could not assess other factors affecting fluid resuscitation. The volume status perceived by the attending physician is an important determinant of fluid resuscitation; however, we did not obtain such data [27]. Moreover, the Less 30 group likely experienced an improved prognosis by avoiding excessive volume overload if they were in a hypervolemic state. The calculation of daily intake and output before the day of sepsis onset was considered, but it was difficult to perform because the time between admission and sepsis onset was different for each patient, and intake and output data were incomplete in several patients. Because of the small number of patients with chronic heart, liver, or kidney diseases, it was not possible to evaluate their effects on fluid resuscitation. However, the proportion of such conditions was at least similar between the two groups. Fifth, we did not provide information on microbiological characteristics or the appropriateness of antibiotics, which could have a significant impact on clinical outcomes, as our study focused on initial resuscitation.

## 5. Conclusions

Patients presenting with sepsis in the general ward were less likely to receive ≥ 30 mL/kg of fluid resuscitation within 3 h, and those with lower body weight or lower systolic blood pressure received ≥30 mL/kg of fluid resuscitation more frequently. Fluid resuscitation ≥30 mL/kg tended to be associated with decreased CRRT use but not with ICU mortality. The timing of blood culture and antibiotic treatment was intricately distributed before and after the onset of sepsis. More research is needed to determine whether fluid resuscitation according to guidelines is effective in patients presenting with sepsis in the general ward and how antibiotic treatment and blood cultures should be performed.

## Figures and Tables

**Table 1:** Baseline characteristics.

Characteristics	All (N = 90)	Less 30 (N = 79)	More 30 (N = 11)	*p* Value
Age, years	66.5 (57.0–75.0)	66.0 (57.0–75.0)	72.0 (61.0–82.0)	0.204
Male	52 (57.8)	48 (60.8)	4 (36.4)	0.192
Body weight, kg	56.7 (50.1–64.4)	57.4 (50.3–67.2)	47.2 (44.9–55.6)	0.005
Comorbidity				
Cardiac disease	12 (13.3)	11 (13.9)	1 (9.1)	>0.999
Chronic respiratory disease	5 (5.6)	5 (6.3)	0	>0.999
Chronic kidney disease	9 (10.0)	8 (10.1)	1 (9.1)	>0.999
Cirrhosis	1 (1.1)	1 (1.3)	0 (0)	>0.999
Solid cancer	41 (45.6)	38 (48.1)	3 (27.3)	0.333
Hematological malignancy	25 (27.8)	23 (29.1)	2 (18.2)	0.721
Neurologic disorder	8 (8.9)	7 (8.9)	1 (9.1)	>0.999
Infection focus				0.646
Pneumonia	26 (28.9)	23 (29.1)	3 (27.3)	>0.999
Urinary tract infection	3 (3.3)	2 (2.5)	1 (9.1)	0.327
Intra-abdominal infection	25 (27.8)	21 (26.6)	4 (36.4)	0.490
Neutropenic fever	17 (18.9)	14 (17.7)	3 (27.3)	0.429
Blood stream infection	9 (10.0)	9 (11.4)	0	0.594
Skin and soft tissue infection	4 (4.4)	4 (5.1)	0	>0.999
Other	6 (6.7)	6 (7.6)	0	>0.999
Hospital length of stay before sepsis onset, days	13.0 (5.0–24.5)	13.0 (5.0–23.0)	12.0 (3.0–27.0)	0.961
SBP, mmHg	80.0 (74.0–85.3)	80.0 (76.0–86.0)	71.0 (62.0–80.0)	0.015
APACHE II	30.0 (25.5–38.0)	31.0 (27.0–39.0)	27.0 (22.0–36.0)	0.173
SpO2 ^a^, %	96.0 (93.0–99.0)	96.0 (92.5–99.0)	98.0 (95.0–99.0)	0.216
Oxygen therapy	47 (52.2)	44 (55.7)	3 (27.3)	0.077
SpO_2_/FiO_2_ ratio^a^	343 (233–461)	336 (233–457)	462 (183–471)	0.191
Antibiotic treatment before sepsis onset	54 (60.0)	48 (60.8)	6 (54.5)	0.749

Data are presented as a median (interquartile range) or number (percentage). ^a^ The SpO_2_ value was not available in 2 patients in the insufficient resuscitation group, and neither was the SpO_2_/FiO_2_ ratio. SBP, systolic blood pressure; APACHE II, Acute Physiology and Chronic Health Evaluation II; SpO_2_, oxygen saturation; FiO_2_, fraction of inspired oxygen.

**Table 2:** Initial sepsis management within 3 h from time zero.

Initial Management	All (N = 90)	Less 30 (N = 79)	More 30 (N = 11)	*p* Value
Total resuscitation fluid volume per body weight, mL/kg	10.5 (6.0–22.0)	9.2 (5.1–17.6)	35.9 (32.8–42.4)	<0.001
Total resuscitation fluid volume, mL	650 (300–1300)	500 (300–1000)	2000 (1500–2000)	<0.001
Vasopressor administration	62 (68.9)	54 (68.4)	8 (72.7)	>0.999
Initial vasopressor				0.242
Norepinephrine	86 (95.6)	76 (96.2)	10 (90.9)	
Vasopressin	1 (1.1)	1 (1.3)	0	
Epinephrine	1 (1.1)	0	1 (9.1)	
Dopamine	2 (2.2)	2 (2.5)	0	
Albumin administration	20 (22.2)	17 (21.5)	3 (27.3)	0.703
Blood cultures	14 (15.6)	12 (15.2)	2 (18.2)	0.679
Newly administered antibiotics	28 (31.1)	22 (27.8)	6 (54.5)	0.090

Data are presented as a median (interquartile range) or number (percentage).

**Table 3:** Clinical outcomes.

Clinical Outcomes	All (N = 90)	Less 30 (N = 79)	More 30 (N = 11)	*p* Value
ICU mortality	39 (43.3)	34 (43.0)	5 (45.5)	>0.999
In-hospital mortality	63 (70.0)	56 (70.9)	7 (63.6)	0.728
ICU length of stay, days	4.0 (2.0–11.0)	5.0 (2.0–11.0)	4.0 (2.0–5.0)	0.621
Hospital length of stay, days	33.0 (21.0–60.3)	35.0 (21.0–61.0)	28.0 (24.0–57.0)	0.542
Mechanical ventilation	49 (54.4)	45 (57.0)	4 (36.4)	0.199
CRRT	22 (24.4)	22 (27.8)	0	0.059

Data are presented as a median (interquartile range) or number (percentage). ICU, intensive care unit; CRRT, continuous renal replacement therapy.

**Table 4:** Factors at the times of sepsis onset associated with ICU mortality.

Variables	Univariable Analysis		Multivariable Analysis	
	Odds Ratio (95% CI)	*p* Value	Odds Ratio (95% CI)	*p* Value
Age, years	0.987 (0.958–1.017)	0.392		
Male	1.923 (0.811–4.558)	0.137		
Body weight, kg	1.036 (0.996–1.077)	0.082	1.036 (0.994–1.081)	0.096
Comorbidity				
Cardiac disease	0.614 (0.171–2.210)	0.456		
Chronic respiratory disease	N/A			
Chronic kidney disease	1.051 (0.263–4.206)	0.943		
Cirrhosis	N/A			
Solid cancer	1.664 (0.713–3.882)	0.239		
Hematologic malignancy	1.038 (0.410–2.631)	0.937		
Neurologic disorder	2.353 (0.526–10.516)	0.263		
Pneumonia	1.175 (0.470–2.938)	0.731		
Hospital length of stay before sepsis onset, days	1.008 (0.988–1.028)	0.457		
SBP, mmHg	1.047 (0.989–1.109)	0.111		
APACHE II	1.090 (1.028–1.155)	0.004	1.090 (1.028–1.156)	0.004
SpO_2_, %	0.976 (0.915–1.040)	0.448		
Oxygen therapy	1.122 (0.487–2.586)	0.787		
SpO_2_/FiO_2_	0.999 (0.995–1.002)	0.404		
Antibiotic treatment before sepsis onset	0.636 (0.271–1.492)	0.299		
Fluid resuscitation of ≥30 mL/kg	1.103 (0.310–3.918)	0.880		
Vasopressor	1.029 (0.418–2.533)	0.951		
Albumin use	0.839 (0.305–2.306)	0.733		
Blood cultures	1.375 (0.439–4.309)	0.585		
Newly administered antibiotics	1.200 (0.489–2.945)	0.691		

Variables with *p* value < 0.1 in univariable analysis were included in the multivariable analysis using the backward elimination method. Fluid resuscitation ≥30 mL/kg was included in the multivariable analysis because it was the primary variable of interest. CI, confidence interval; SBP, systolic blood pressure; APACHE II, Acute Physiology and Chronic Health Evaluation II; SpO_2_, oxygen saturation; FiO_2_, fraction of inspired oxygen.

**Table 5:** Factors associated with fluid resuscitation ≥30 mL/kg.

Variables	Univariable Analysis		Multivariable Analysis	
	Odds Ratio (95% CI)	*p* Value	Odds Ratio (95% CI)	*p* Value
Age, years	1.041 (0.984–1.102)	0.160		
Male	0.369 (0.100–1.366)	0.136		
Body weight, kg	0.891 (0.813–0.976)	0.013	0.896 (0.811–0.989)	0.030
Comorbidity				
Cardiac disease	0.618 (0.072–5.318)	0.661		
Chronic respiratory disease	N/A			
Chronic kidney disease	0.888 (0.100–7.865)	0.915		
Cirrhosis	N/A			
Solid cancer	2.472 (0.610–10.006)	0.205		
Hematologic malignancy	0.541 (0.108–2.699)	0.454		
Neurologic disorder	1.029 (0.114–9.257)	0.980		
Pneumonia	0.913 (0.222–3.751)	0.900		
Hospital length of stay before sepsis onset, days	0.998 (0.968–1.030)	0.919		
SBP, mmHg	0.899 (0.831–0.972)	0.008	0.916 (0.846–0.992)	0.031
SpO_2_, %	1.024 (0.919–1.141)	0.670		
Oxygen therapy	0.298 (0.074–1.209)	0.090		
SpO_2_/FiO_2_	1.002 (0.997–1.008)	0.375		
Antibiotic treatment before sepsis onset	0.775 (0.218–2.759)	0.694		
Vasopressor	1.235 (0.302–5.052)	0.769		
Albumin use	1.368 (0.327–5.722)	0.668		
Blood cultures	1.241 (0.238–6.465)	0.798		
Newly administered antibiotics	3.109 (0.860–11.235)	0.084		

Variables with *p* value < 0.1 in univariable analysis were included in the multivariable analysis using the backward elimination method. CI, confidence interval; SBP, systolic blood pressure; SpO_2_, oxygen saturation; FiO_2_, fraction of inspired oxygen.

## Data Availability

The datasets used and/or analyzed during the current study are available from the corresponding author upon reasonable request.

## Supplementary Materials

The following supporting information can be downloaded at: https://www.mdpi.com/article/10.3390/life15010124/s1, Table S1. Time to each sepsis management from time zero. Table S2. Time distribution of blood cultures. Table S3. Time distribution of antibiotic administration.
