# Supplementary material for: Fluid Resuscitation and Initial Management in Patients Presenting with Sepsis in the General Ward
Source: Life (Basel). 2025 Jan 18;15(1):124. doi: 10.3390/life15010124 (PMC11767154; doi:10.3390/life15010124)
Supplement: Supplementary file 1 [file life-15-00124-s001.zip › life-3386319-supplementary.pdf]

Table S1. Time to each sepsis management from time zero

Management	All (N = 90)	Less 30 (N = 79)	More 30 (N = 11)	*P* value
Time to fluid resuscitation, min[a]	1 (0-30)	2 (0-40)	0 (0-12)	0.068
Time to vasopressor, min	120.0 (54.5-249.8)	123.0 (57.0-275.0)	90.0 (32.0-190.0)	0.160
Time to blood culture, min[b]	237.0 (89.0-519.0)	241.0 (89.3-521.3)	146.0 (51.0-)	0.322
Time to newly administered antibiotics, min[c]	159.5 (55.0-278.5)	179.5 (79.5-394.8)	59.5 (18.8-142.3)	0.011

Data are presented with a median (interquartile range).

[a] Time to fluid resuscitation was not available in 4 patients in the insufficient resuscitation group. Three patients did not receive fluid resuscitation, and 1 received fluid resuscitation within 3 h from time zero without an exact start time.

[b] Time to blood culture was calculated in 32 patients and 3 patients in the insufficient resuscitation group and the sufficient resuscitation group, respectively.

[c] Time to newly administered antibiotics was calculated in 40 patients and 8 patients in the insufficient resuscitation group and in the sufficient resuscitation group, respectively.

Table S2. Time distribution of blood cultures

	All (N = 90)	Less 30 (N = 79)	More 30 (N = 11)	*P* value
Before the day of sepsis onset	20 (22.2)	17 (21.5)	3 (27.3)	0.705
On the day of sepsis onset	63 (70.0)	56 (70.9)	7(63.6)	0.724
Before time zero	35 (38.9)	30 (38.0)	5(45.5)	
After time zero	28 (31.1)	26 (32.9)	2(18.2)	
After the day of sepsis onset	6 (6.7)	5 (6.3)	1(9.1)	0.558

Data are presented with a number (percentage). In one patient in the insufficient resuscitation group, blood cultures were not performed for two days before and after the day of sepsis onset.

Table S3. Time distribution of antibiotic administration

	All (N = 90)	Less 30 (N = 79)	More 30 (N = 11)	*P* value
Continuation of the same antibiotics	11 (12.2)	11 (13.9)	0 (0)	0.217
Newly administered antibiotics	79 (87.8)	68 (86.1)	11 (100)	0.217
Before time zero	31 (34.4)	28 (35.4)	3 (27.3)	
After time zero	48 (53.3)	40 (50.6)	8 (72.7)	

Data are presented with a number (percentage).
